# The impact of concurrent bacterial lung infection on immunotherapy in patients with non-small cell lung cancer: a retrospective cohort study

**DOI:** 10.3389/fcimb.2023.1257638

**Published:** 2023-08-29

**Authors:** Qiang Cao, Xinyan Wu, Yuquan Chen, Qi Wei, Yanwei You, Yi Qiang, Guangzhu Cao

**Affiliations:** ^1^Department of Earth Sciences, Kunming University of Science and Technology, Kunming, China; ^2^School of Medicine, Macao University of Science and Technology, Macao, Macao SAR, China; ^3^College of Veterinary Medicine, Sichuan Agricultural University, Chengdu, China; ^4^Institute of Medical Information/Library, Chinese Academy of Medical Sciences, Beijing, China; ^5^Division of Sports Science & Physical Education, Tsinghua University, Beijing, China

**Keywords:** non-small cell lung cancer, bacterial lung infection, microbial infection, lung cancer, retrospective cohort study, and immunotherapy

## Abstract

**Objective:**

To find out how bacterial lung infections (BLI) affect the effectiveness of therapy and the rate of pneumonia caused by pneumonia related to checkpoint inhibitors (CIP) in patients with non-small cell lung cancer (NSCLC) who are getting immunotherapy with checkpoint inhibitors (ICIs).

**Patients and methods:**

507 NSCLC patients who received at least two ICI treatments between June 2020 and December 2022 at the Affiliated Hospital of Kunming University of Science and Technology(AHKUST) were included in a retrospective cohort study. Based on whether there was a concurrent BLI diagnosis from high-resolution CT scans of the chest, the patients were divided into two groups: 238 in the NSCLC with BLI group (NSCLC-BLI group), and 269 in the NSCLC alone group. The collected therapeutic outcome measures included the objective response rate (ORR), progression-free survival (PFS), overall survival (OS), and the incidence rate of CIP. We analyzed the effect of BLI on the therapeutic efficacy of ICI treatment and the incidence rate of CIP in NSCLC patients.Inclusion criteria based on NSCLC patients staged I to IV according to the 8th edition of the International Association for Lung Cancer Research (IASLC)

**Results:**

The NSCLC-BLI group showed superior ORR to the NSCLC group when treated with ICIs. Multifactorial logistic regression and Cox analyses, adjusted for confounders, identified BLI as an independent positive prognostic factor for ORR (HR=0.482, 95%CI: 0.391-0.550; P<0.001) and PFS (HR=0.619; 95%CI: 0.551-0.771; P<0.001). No correlation between BLI and OS was found. Out of 26 cases of CIP, 12 were in the NSCLC-BLI group and 14 in the NSCLC group, with no significant difference in incidence (P=0.145).

**Conclusion:**

NSCLC patients with BLI receiving ICI treatment show superior ORR and PFS compared to NSCLC alone without an increased CIP risk, positioning BLI as a predictive factor for improved outcomes in NSCLC patients receiving ICIs. However, the study has limitations including its retrospective nature and lacking data on BLI bacteria types and levels, which could influence therapy outcomes.

## 1 Introduction

Lung cancer (CA) is a prevalent malignancy with high incidence and mortality rates worldwide (Hakozaki et al., 2020; Cortellini et al., 2021b). NSCLC is diagnosed in 85 percent of lung cancer cases (Nyein et al., 2022; Xi et al., 2022). Bacterial lung infection (BLI) refers to a disease caused by microbial infection in the lungs, which induces inflammation and alters the lung microbiome environment (Oliva and Terrier, 2021; Vallianou et al., 2023). In particular, BLI, as referred in this study, involves a variety of bacterial pathogens such as Streptococcus pneumoniae, Haemophilus influenzae, and Pseudomonas aeruginosa, all known for their capacity to colonize the respiratory tract and trigger infection.The World Health Organization’s 2021 Global Cancer Report indicated that 60% to 80% of NSCLC patients have concurrent BLI (He et al., 2021). A large body of evidence demonstrates a close link between the pathogenesis of BLI and NSCLC (Cao et al., 2022a; Hallin et al., 2022). The inflammatory and immune microenvironments induced by BLI are closely associated to the development and progression of NSCLC (Takada et al., 2022; Tang et al., 2023). Immunotherapy has made significant strides in the treatment of NSCLC in recent years, particularly with ICIs represented by inhibitors of the programmed cell death protein 1 (PD-1)/programmed cell death protein ligand 1 (PD-L1) pathway, which have substantially enhanced NSCLC patients’ survival and prognosis (Yan et al., 2022; You et al., 2023). However, a substantial number of patients do not benefit from ICIs, and there is a risk of adverse effects related to immunotherapy, especially the incidence of checkpoint inhibitor-related pneumonia (CIP) in NSCLC patients, which is significantly higher than in patients with other types of tumors (Suresh et al., 2019; Cao et al., 2022c). As a common comorbidity in NSCLC patients, the characteristic inflammation and immune micro-environment caused by BLI may reduce the therapeutic efficacy of ICIs in patients with NSCLC (He et al., 2020; Cao et al., 2022b). Lin etal’s study (Lin et al., 2021) shown that a significant correlation between BLI and the incidence of CIP. Conversely, Kovaleva etal’s study (Kovaleva et al., 2020) considers BLI as a favorable prognostic factor for NSCLC. Therefore, it is of great clinical significance to explore the impact of BLI on immunotherapy for NSCLC. Accordingly, the primary focus of this research is to comprehensively delineate the consequential implications of concurrent bacterial lung infections on the therapeutic efficacy of immune checkpoint inhibitors in managing non-small cell lung cancer, in addition to evaluating its potential effect on the prevalence of checkpoint inhibitor-induced pneumonia. This study investigates the influence of BLI on the clinical efficacy of ICIs and the occurrence of CIP in NSCLC patients through a retrospective cohort study.The foremost aim of this investigation is to explicate the ramifications of bacterial lung infections on the therapeutic effectiveness of checkpoint inhibitors within the context of patients afflicted with non-small cell lung cancer. Moreover, this research endeavors to analyze the potential impact on the incidence rate of checkpoint inhibitor-associated pneumonia.

## 2 Patients and methods

### 2.1 Study subjects and grouping

This study uses a retrospective cohort method. Case information of NSCLC patients who received ICIs treatment at the AHKUST from June 2020 to December 2022 was collected.

Inclusion criteria: ① Pathologically diagnosed as NSCLC; ② NSCLC patients staged I to IV according to the 8th edition of the International Association for Lung Cancer Research (IASLC); ③ NSCLC patients who have received at least 2 treatments with PD-1 or PD-L1 inhibitors; ④ Absence of EGFR, ALK, ROS1 mutations.

Exclusion criteria: ① No evaluable lesion; ② Inability to obtain efficacy evaluation information.

Based on whether concurrent BLI was diagnosed by the 2021 Global Initiative for Chronic Obstructive Lung Disease (GOLD) or high-resolution CT of the chest, patients were divided into NSCLC with BLI group (NSCLC-BLI group) and pure NSCLC group (NSCLC group). This study was approved by the Ethics Committee of Kunming University of Science and Technology (KUST20200110221380).

### 2.2 Baseline indicators

Using a uniform questionnaire, patient age, gender, smoking history, performance status (PS) score, underlying medical history, histopathological type, clinical staging, immunotherapy plan, number of immunotherapy lines, tumor mutation burden (TMB), the AHKUST’s electronic medical record system and the Kunming Medical Insurance System’s PD-L1 expression results were obtained.

### 2.3 Efficacy evaluation indicators and CIP incidence

The primary outcome measure for this investigation is PFS, with OS, ORR, and incidence rate of CIP as secondary outcome measures. All patients, including those with Complete Response (CR), Progressive Disease (PD), Partial Response (PR), and Stable Disease (SD), are evaluated according to the Response Evaluation Criteria In Solid Tumours (RECIST) for the efficacy of therapy. ORR is calculated as the number of (CR+PR) cases divided by the total number of cases. PFS refers to the period from the initial treatment with PD-1 or PD-L1 inhibitors until the progression of the disease or death due to any cause. OS refers to the period from the initial treatment with PD-1 or PD-L1 inhibitors until death due to any cause. The diagnosis, differential diagnosis, and grading of CIP are conducted in reference to the “National Cancer Information Community guidelines.”

### 2.4 Follow-up visit

Patients were followed up by reviewing outpatient and hospital records and by telephone inquiry. A unified follow-up survey form was used to record patients’ disease progression, CIP occurrence, and survival prognosis. The last follow-up date was December 25, 2022.

### 2.5 Statistical analysis

Data enumerations were articulated through numerical representation (%), wherein both χ2 testing methodology and Fisher’s examination were employed to evaluate intergroup variance. Independent sample continuous variables underwent comparative analysis using the t-test. Calculations for progression-free survival (PFS) and overall survival (OS) were performed through the utilization of the Kaplan-Meier estimation technique, with distinctions subsequently appraised through the Log-rank testing approach. The correlational assessment between variables and objective response rate (ORR) was performed via logistic regression analysis. Simultaneously, the relationship between variables and PFS and OS was examined using Cox proportional hazards statistical regression. Tthe hazard ratio (HR) and the 95% confidence interval (CI) were ascertained. For multifactor analysis, the first step was to include variables other than BLI in the regression model and use backward selection to screen potential confounders, retaining statistically significant variables as covariates. The second step was to include all covariates along with BLI in the regression model to analyze the association of BLI with ORR, PFS, and OS. The R 4.0 software was used for all statistical analyses. A P-value for a two-sided test less than 0.01 was statistically significant.

## 3 Results

### 3.1 Patient baseline characteristics

As shown in **Table 1**, this study included a total of 507 NSCLC patients, with 238 in the NSCLC-BLI group and 269 in the NSCLC group. Compared to the NSCLC group, the NSCLC-BLI group had a higher proportion of male patients, those with a smoking history, and patients on immunotherapy monotherapy (P<0.01). The incidence of disease progression was considerably lower in the NSCLC-BLI group (P<0.01), and the average age was significantly older in the NSCLC-BLI group than in the NSCLC group. In terms of PS score, histological type, and TNM staging, there was no statistically significant difference (P>0.01) between the two groups. PD-L1 expression did not differ significantly between the two groups on a statistical basis; 34.7% of cases (176 patients) had PD-L1 expression more than 50% in tumour cells. The proportion of high and medium TMB expression in the NSCLC-BLI group was higher than in the NSCLC group. Of the 507 patients enrolled, 26 developed CIP, with 12 cases (5.0%) in the NSCLC-BLI group and 14 cases (5.2%) in the NSCLC group, showing no significant statistical difference (P=0.145).

**Table 1:** Comparison of baseline clinical characteristics between the NSCLC-BLI and NSCLC groups.

variable	Total cases(*n*=507)	NSCLC-BLI(*n*=238)	NSCLC(*n*=269)	*P*
**Age/Year**	60.4 ± 8.2	66.1 ± 7.2	56.4 ± 9.5	
**Sex(%)**				<0.001
**Male**	372(73.4%)	190(79.8%)	182(67.65%)	
**Female**	135(26.6%)	48(20.2%)	87(32.34%)	
**Smoking history**				<0.001
**Smoking**	363(71.6%)	192(64.4%)	171(63.6%)	
**Never smoked.**	144(28.4%)	46(35.6%)	98(36.4%)	
**PS Rating**				0.375
**0~1**	380(74.9%)	181(76.0%)	199(73.9%)	
**2~3**	127(25.1%)	57(24.0%)	70(26.1%)	
**Histological type**				0.235
**Squamous cancer**	337(66.5%)	162(68.1%)	175(65.1%)	
**Non-squamous cancer**	170(33.5%)	76(31.9%)	94(34.9%)	
**TNM staging**				0.026
**I**	84(16.6%)	39(16.4%)	45(16.7%)	
**II**	219(43.2%)	102(42.9%)	117(43.5%)	
**III**	100(19.7%)	49(20.6%)	51(19.0%)	
**IV**	104(20.5%)	48(20.1%)	56(20.8%)	
**Treatment programme**				<0.001
**Immunological monotherapy**	98(19.3%)	60(25.2%)	38(14.1%)	
**Combination chemotherapy**	256(50.5%)	104(43.7%)	152(56.5%)	
**Combined anti-angiogenic therapy**	66(13.0%)	31(13.0%)	35(13.0%)	
**Combination radiotherapy**	87(17.2%)	43(18.1%)	44(16.4%)	
**PD-L1 expression**				<0.001
**Negatives**	180(35.5%)	87(36.5%)	93(34.6%)	
**1%~49%**	131(25.8%)	50(21.0%)	81(30.1%)	
**≥50%**	196(38.7%)	101(42.5%)	95(35.3%)	
**TMB**				<0.001
**High**	233(46.0%)	116(48.7%)	117(43.5%)	
**Middle**	230(45.4%)	115(48.3%)	115(42.7%)	
**Low**	44(8.6%)	7(3.0%)	37(13.8%)	
**CIP**				0.145
**Yes**	26(5.1%)	12(5.0%)	14(5.2%)	
**No**	481(94.9%)	226(95.0%)	255(94.8%)	
**End**				
**Progress**	171(33.7%)	69(29.0%)	102(37.9%)	0.007
**Die**	436(66.3%)	169(71.0%)	267(62.1%)	0.531

### 3.2 Logistic regression analysis of factors influencing the ORR of NSCLC patients treated with ICIs

The ORR of NSCLC-BLI group treated with ICIs was better than that of the NSCLC group (51.9% vs 32.8%; P=0.003). As shown in **Table 2**, univariate analysis showed that factors influencing the ORR of NSCLC patients treated with ICIs included being male, smoking, having combined BLI, squamous carcinoma, treatment line number, and combined chemotherapy. Adjusted for confounding factors, the multivariate logistic regression analysis revealed that combined BLI, squamous carcinoma, and combined chemotherapy are independent factors influencing the ORR of NSCLC patients treated with ICIs.

**Table 2:** Analysis of factors influencing the ORR of NSCLC patients treated with ICIs using logistic regression.

Variable	One-way analysis of variance		Multifactorial analysis	
	*HR* (95%CI)	*P*	*HR* (95%CI)	*P*
**Age>60**	1.252(1.131-1.306)	0.517	–	–
**Male**	1.329(1.280-1.415)	<0.001	–	–
**Smoking**	1.420(1.351-1.592)	<0.001	–	–
**Combined BLI**	0.476(0.435-0.609)	<0.001	0.482(0.391-0.550)	<0.001
PS Rating				
**0~1**	1	–	–	–
**2~3**	1.382(1.198-1.510)	0.482	–	–
**Squamous cancer**	0.358(0.209-0.476)	<0.001	0.403(0.338-0.517)	<0.001
PD-L1 expression				
**Negatives**	1	–	–	–
**1%~49%**	1.242(1.125-1.401)	0.392	–	–
**≥50%**	1.148(1.062-1.285)	0.504	–	–
Number of immunotherapy lines				
**≥2**	1		–	–
**1**	0.302(0.204-0.419)	<0.001	–	–
Treatment programme				
**Immunological monotherapy**	1	–	1	–
**Combination chemotherapy**	0.336(0.295-0.402)	<0.001	0.358(0.224-0.458)	<0.001
**Combination radiotherapy**	0.735(0.682-0.893)	0.451	0.802(0.733-0.902)	0.281
**Combined anti-angiogenic therapy**	1.622(1.526-1.708)	0.508	1.320(1.224-1.497)	0.792

### 3.3 Cox regression analysis of prognostic factors for NSCLC patients treated with ICIs

The median PFS in the NSCLC-BLI group receiving ICIs treatment was significantly better than the NSCLC group (12.5 months vs 7.8 months, P=0.002). As shown in **Table 3**, univariate analysis shows that the factors affecting PFS of NSCLC patients undergoing ICIs treatment include concurrent BLI, male gender, smoking, squamous cell carcinoma, and first-line treatment status. Multivariate Cox regression analysis after adjusting for confounding factors showed that the treatment line number being the first-line and concurrent BLI are independent prognostic factors affecting the PFS of NSCLC patients receiving ICIs treatment.

**Table 3:** Cox regression analysis of variables impacting NSCLC patients treated with ICIs in relation to PFS.

Variable	One-way analysis of variance		Multifactorial analysis	
	*HR* (95%CI)	*P*	*HR* (95%CI)	*P*
**Age>60**	1.258 (1.116-1.325)	0.356	–	–
**Male**	1.410 (1.332-1.518)	<0.001	–	–
**Smoking**	1.625 (1.482-1.709)	<0.001	–	–
**Combined BLI**	0.338 (0.213-0.461)	<0.001	0.619 (0.524-0.791)	<0.001
PS Rating				
**0~1**	1	–	–	–
**2~3**	1.604 (1.472-1.739)	0.882	–	–
**Squamous cancer**	0.632 (0.403-0.728)	<0.001	–	–
PD-L1 expression				
**Negatives**	1	–	–	–
**1%~49%**	1.299 (1.142-1.390)	0.583	–	–
**≥50%**	1.258 (1.132-1.347)	0.277	–	–
Treatment programme				
**Immunological monotherapy**	1	–	–	–
**Combination chemotherapy**	0.751 (0.669-0.802)	0.611	–	–
**Combination radiotherapy**	1.206 (1.114-1.257)	0.352	–	–
**Combined anti-angiogenic therapy**	1.547 (1.429-1.616)	0.329	–	–
Number of immunotherapy lines				
**≥2**	1	–	1	–
**1**	0.320 (0.274-0.458)	<0.001	0.392 (0.288-0.463)	<0.001

In terms of median OS, there was no statistically significant distinction between NSCLC-BLI and NSCLC. (19.5 months vs 19.7 months, P=0.841). As delineated in **Table 4**, the findings from the multivariate Cox proportional hazards regression analysis, following the adjustment for potential confounding variables, indicates that the position of the treatment line as the primary one substantially influences the OS of patients with NSCLC undergoing treatment with ICIs. Notably, the analysis did not reveal a statistically significant correlation between BLI and the OS of these patients.

**Table 4:** The outcomes of a Cox regression analysis of OS-related factors impacting NSCLC patients treated with ICIs.

variable	One-way analysis of variance		Multifactorial analysis	
	*HR* (95%CI)	*P*	*HR* (95%CI)	*P*
**Age>60**	1.352 (1.206-1.459)	0.340	–	–
**Male**	1.570 (1.299-1.826)	0.216	–	–
**Smoking**	1.601 (1.523-1.739)	0.382	–	–
**Combined BLI**	0.612(0.508-0.719)	0.597	0.739 (0.651-0.843)	0.816
PS Rating				
**0~1**	1	–	–	–
**2~3**	1.429 (1.306-1.518)	0.659	–	–
**Squamous cancer**	0.832 (0.770-0.893)	0.516	–	–
PD-L1 expression				
**Negatives**	1	–	–	–
**1%~49%**	1.318(1.119-1.534)	0.338	–	–
**≥50%**	1.329(1.227-1.410)	0.826	–	–
Treatment programme				
**Immunological monotherapy**	1	–	–	–
**Combination chemotherapy**	0.829 (0.726-0.891)	0.401	–	–
**Combination radiotherapy**	1.419(1.352-1.510)	0.329	–	–
**Combined anti-angiogenic therapy**	1.262 (1.198-1.306)	0.135	–	–
Number of immunotherapy lines				
**≥2**	1	–	1	–
**1**	0.525 (0.446-0.627	<0.001	0.526 (0.448-0.619)	<0.001

## 4 Discussion

In contemporary times, substantial advancements have been made in the realm of lung cancer treatment by the prominent use of ICIs, a representative form of immunotherapies. These developments have considerably enhanced the survival rates and prognostic outlook of patients with NSCLC who exhibit a lack of driver gene mutations. Budisan etal’s study (Budisan et al., 2021) shown that BLI is an independent risk factor for the development of NSCLC. However, Remon’s study (Remon et al., 2020) pointed out that there’s not enough evidence-based medical evidence about whether the characteristic inflammation and immune micro-environment of BLI would impact the efficacy and safety of ICIs in lung cancer patients. In our retrospective cohort study of NSCLC patients receiving ICIs treatment, after adjusting for confounding factors, multifactorial Logistic analysis showed that concomitant BLI is a protective factor influencing the ORR after NSCLC patients receive ICIs treatment (HR=0.482,95%CI:0.391~0.550;P<0.001), BLI may also be a protective factor for PFS (HR=0.619;95%CI:0.524~0.791;P<0.001), whereas BLI has no apparent correlation with OS, which is consistent with Ding etal’s study (Ding et al., 2023) shown that NSCLC patients with concurrent BLI may have better efficacy from immunotherapy.

NSCLC is a frequent comorbidity and cause of mortality associated with BLI (Gomes et al., 2020; Russano et al., 2023). Moik etal.’s study (Moik et al., 2020) illuminates the position of the BLI as a robust, independent prognosticator for adverse outcomes in NSCLC. One plausible explanation for this correlation is the common incidence of BLI among elderly lung cancer patients who typically present with compromised pulmonary function, thereby influencing the ideal therapeutic course for lung cancer.In our research sample, it was observed that the median age of NSCLC patients afflicted with BLI noticeably exceeded that of patients with NSCLC in isolation. Intriguingly, however, the former group demonstrated superior ORR and PFS following ICIs treatment in comparison to those with NSCLC alone. This observation leads to the hypothesis that ICIs treatment may potentially offset the negative implications that BLI holds for the prognosis of patients diagnosed with NSCLC (Boesch et al., 2021; Prasetya et al., 2021; Xie et al., 2021). This study observed that concurrent BLI significantly influenced the therapeutic outcomes in NSCLC patients undergoing ICIs. It is noteworthy that patients classified in the NSCLC with BLI group exhibited a superior ORR and extended PFS in comparison to the NSCLC group. This result suggests that the simultaneous presence of BLI may contribute positively to the therapeutic response to ICIs. These empirical findings cohere with our initial suppositions and highlight the prospective predictive merit of BLI concerning the outcomes of ICI therapy.Despite the encouraging outcomes, the OS rate did not evince a significant variance between the two cohorts. This area remains an intriguing venue for future investigation to discern potential factors influencing OS in NSCLC patients with co-existing BLI. Moreover, the incidence of CIP was not significantly different between the two cohorts. This observation holds significance given the apprehensions previously raised in academic discourses about the potentially amplified risk of CIP in NSCLC patients concurrently afflicted with BLI. Our results emphasize the potential of BLI as a favorable prognostic index for ORR and PFS in NSCLC patients subjected to ICI therapy. These empirical outcomes bear substantial implications for the clinical milieu. It appears that NSCLC patients co-diagnosed with BLI might anticipate superior therapeutic outcomes upon ICI treatment, characterized by enhanced ORR and PFS. This indication suggests the potential for patient stratification in NSCLC based on the presence of BLI, enhancing the predictive accuracy for ICI treatment outcomes.This insight could urge clinicians to accord meticulous attention to BLI in their NSCLC patient cohort and contemplate it as a potential determinant in tailoring immunotherapy protocols. It is paramount to acknowledge that the concurrent existence of BLI did not elevate the risk of CIP onset, which further corroborates the role of BLI in managing NSCLC. Some studies suggest that certain bacteria may have immunomodulatory properties, and could potentially enhance the immune response against cancer cells. This effect could be partly responsible for the improved ORR and PFS seen in NSCLC patients with BLI receiving ICIs.On the other hand, the inflammatory response to BLI could lead to the recruitment and activation of various immune cells in the lung, potentially increasing the ‘visibility’ of cancer cells to the immune system. This could enhance the anti-tumor effect of ICIs, contributing to the superior therapeutic outcomes observed in our study.

The impact of BLI on the lung cancer tumor micro-environment and the effectiveness of ICIs treatment has important implications for the clinical treatment of NSCLC, but previous research on this topic has been limited. Tan etal’s study (Tan et al., 2021) confirmed that the characteristic immune dysfunction and inflammation mechanism of BLI may play a crucial role in the immune micro-environment of lung cancer development. Braverman etal’s study (Braverman et al., 2022) pointed out the increase in CD4+Th1, CD4+Th17 cells in BLI lung tissue, which play a vital role in maintaining BLI’s airway inflammation. Kuen etal’s study (Kuen et al., 2020) found in a simulated BLI-like inflammatory lung cancer mouse model that the BLI inflammatory environment promotes Th17 cell proliferation, and IL-17 regulates tumor cell proliferation and promotes angiogenesis. Arcadu etal’s study (Arcadu et al., 2022) shows that elevated serum Th17 related cell factors are independent risk factors for the occurrence of lung cancer with BLI. Jin etal’s study (Jin et al., 2022) shown that an increased proportion of Th1 and Th17 cells in lung cancer tissue is a marker of poor prognosis for lung cancer. Under the BLI inflammatory environment, Treg cells gather in the lungs to inhibit inflammation, Treg cells inhibit the proliferation of effector T cells and play a key role in maintaining self-immune tolerance, which could lead to persistent infection and tumor development (Zullo et al., 2021; Qiang et al., 2022; Yuan et al., 2023). Jiang etal’s study (Shi et al., 2022) found a negative correlation between Foxp3+Tregs cell infiltration in the tumor matrix and lung cancer prognosis. During the development of BLI, there is an increase in the proportion of CD8+PD-1+T lymphocytes in BLI lung tissue, mainly characterized by T cell functional exhaustion, weakening the body’s anti-infection and anti-tumor effects. Shi etal’s study (Shi et al., 2022) found that the number of PD1+CD8+ and TIM3+CD8+T lymphocytes in the tumor micro-environment of NSCLC patients with BLI increased significantly compared to patients with pure NSCLC, the anti-tumor immune function of exhausted CD8+T cells is impaired, the secretion of cell factors such as IFN-γ and TNF-α is reduced, and the expression of inhibitory receptors increases. This suggests that the increase in exhausted CD8+T lymphocytes in the BLI micro-environment may be closely related to the occurrence and progression of tumors. Our study found that NSCLC patients with concurrent BLI had better ORR and PFS after immunotherapy, consistent with previous studies (Cortellini et al., 2021a; Ryssel et al., 2022; Zhang et al., (2022)). The mechanism for this phenomenon may be that the characteristic tumor micro-environment of BLI is more suitable for PD-1/PD-L1 pathway inhibitors to work, thereby reversing T lymphocyte exhaustion in tumor tissues, restoring T lymphocyte activity, and better exerting anti-tumor effects. Our study shows that, compared to the NSCLC group, the NSCLC-BLI group showed a trend towards high PD-L1 expression (42.5% vs 35.3%), with a statistical difference (<0.001). In subgroup analysis, PD-L1≥50% patients with concurrent BLI showed a trend towards longer PFS. In the investigation undertaken by us, there emerged no substantial discrepancy in the expression of PD-L1 between the cohorts of NSCLC-BLI and NSCLC. It is universally acknowledged within the medical research community that the expression of PD-L1 functions as a highly reliable predictive biomarker that corresponds with the efficacy of immunotherapy. This is particularly discernible in the context of treatments that incorporate inhibitors targeting PD-1 or PD-L1. Between higher levels of PD-L1 expression and increased responsiveness to immunotherapy in NSCLC patients. Our observation of no significant difference in PD-L1 expression between the two groups, despite differences in ORR and PFS, might suggest that other factors, such as the concurrent BLI and its associated inflammatory and immune micro-environment, could be playing a pivotal role in shaping the response to ICI treatment. Despite our study revealing a significant correlation between concurrent BLI and both the ORR and PFS, there was no significant correlation found between BLI and OS. That’s probably because the inflammatory and immune responses elicited by BLI, which may enhance ORR and PFS, could lose their efficacy over time, not extending the overall survival period. This is plausible given that the chronic inflammatory environment could lead to immunosuppression, negating the positive effect of immune activation on the survival of NSCLC patients. Furthermore, bacterial infections often come with an increased risk of complications and other co-morbidities that might have a detrimental effect on the patient’s overall survival.

CIP is a relatively common pulmonary toxicity reaction to ICIs, and previous studies have shown that it is related to the occurrence of BLI. This study found that concomitant BLI does not increase the risk of CIP after immunotherapy (P=0.145). However, Lin etal’s study (18) suggests that concomitant BLI is an independent predictor of an increased incidence of CIP. But another study on BLI and NSCLC patients receiving ICIs treatment showed that BLI does not increase the risk of CIP. The inconsistency in the above research results may be related to the small sample size and the inclusion of different degrees of BLI severity (Tian et al., 2018; Chen et al., 2021; He et al., 2022). Therefore, whether concurrent BLI increases the risk of CIP in NSCLC patients receiving ICIs treatment needs to be confirmed by large sample prospective studies.

This study’s strength rests in large sample size and the use of multivariate analysis methods, controlling for potential confounding factors that might influence the study results. By adjusting for other potential influencing factors, the study results can more accurately assess the impact of concurrent BLI on the efficacy of ICIs treatment and the incidence of CIP in NSCLC patients. This study has some shortcomings. This study is a retrospective cohort, which limits the representativeness of the subjects and the generalizability of the results. At the same time, most of the cases in this study were diagnosed with BLI based on high-resolution chest CT, which may be affected by subjective judgments between observers and leads to selectivity bias, and the correlation between the severity of BLI airflow obstruction and ICIs efficacy was not observed. Follow-up studies will further carry out multi-center, large sample prospective cohort studies to clarify the immune micro-environment characteristics of NSCLC patients with concurrent BLI and its association with efficacy and safety, accumulating evidence-based medical evidence for further optimizing the advantage population for lung cancer immunotherapy.

## 5 Conclusion

This study confirms that BLI is an independent predictive factor for the efficacy of ICIs treatment in NSCLC patients. It demonstrates that the ORR and PFS of NSCLC patients with concurrent BLI receiving ICIs treatment are significantly better than those of NSCLC patients alone. In this study, concurrent BLI did not increase the risk of CIP occurrence in patients receiving ICIs treatment, providing evidence-based medical evidence for NSCLC patients with concurrent BLI receiving ICIs treatment. This indicates that BLI is a predictive factor for obtaining better efficacy in NSCLC patients receiving ICIs treatment. It provides evidence-based medical evidence for NSCLC patients with concurrent BLI receiving ICIs treatment.

## Data availability statement

The original contributions presented in the study are included in the article/supplementary material. Further inquiries can be directed to the corresponding authors.

## Ethics statement

The patient in our research has signed the informed consent. This study was designed in accordance with the Declaration ofHelsinki and approved by the ethics committee of Kunming University of Science and Technology. Approval number:KUST20200110221380.

## Author contributions

Conceptualization: QC, XW, YC, QW, YY, YQ, and GC. Methodology: QC, XW, YC, QW, YY, YQ, and GC. Validation: QC, XW, YC, QW, YY, YQ, and GC. Formal analysis: QC, XW, YC, QW, YY, YQ, and GC. Investigation: QC, XW, YC, QW, YY, YQ, and GC. Resources: QC, XW, YC, QW, YY, YQ, and GC. Data curation: QC, XW, YC, QW, YY, YQ, and GC. Writing - original draft: QC, XW, YC, QW, YY, YQ, and GC. Writing - review and Editing: QC, XW, YC, QW, YY, YQ, and GC. Supervision: QC, XW, YC, QW, YY, YQ, and GC. Project administration: QC, XW, YC, QW, YY, YQ, and GC. All authors contributed to the article and approved the submitted version.
